# Case Report: Moderate-to-severe paravalvular leak regurgitation after recurrent prosthetic valve endocarditis in a patient with a double-chambered right ventricle associated with a restricted membranous ventricular septal defect

**DOI:** 10.3389/fcvm.2025.1558686

**Published:** 2025-05-14

**Authors:** Cosimo Sacra, Antonio Totaro, Giuseppe Triggiani, Andrea Romano, Pasquale Astore, Chiara Galluccio, Eustaquio Maria Onorato

**Affiliations:** ^1^ Interventional Cardiology Unit, Responsible Research Hospital, Campobasso, Italy; ^2^ Department of Medicine and Health Sciences “V. Tiberio”, University of Molise, Campobasso, Italy; ^3^ University Cardiology Department, IRCCS Galeazzi-Sant’Ambrogio Hospital, Milan, Italy

**Keywords:** infective endocarditis, surgical aortic valve replacement, paravalvular leak regurgitation, paravalvular leak closure, double-chambered right ventricle, membranous ventricular septal defect

## Abstract

**Background:**

Managing aortic paravalvular leak (PVL) regurgitation following multiple surgical aortic valve replacements (SAVRs) due to recurrent infective endocarditis (IE) presents significant clinical challenges.

**Case summary:**

A 46-year-old woman with a history of severe aortic regurgitation and an asymptomatic membranous ventricular septal defect underwent SAVR with a bioprosthetic aortic valve (Perimount 23 mm) in 2005. Concomitantly, a double-chambered right ventricle was diagnosed. Ten years later, due to recurrent IE, another bioprosthetic valve replaced the previous valve (Magna Ease #25). In 2018, she developed sepsis from *Bordetella hinzii* endocarditis, leading to a third SAVR in 2019, this time with a mechanical aortic valve (On-X® #23). In 2024, two-dimensional transesophageal echocardiography (TEE) revealed moderate-to-severe PVL regurgitation near the right coronary cusp. After a multidisciplinary evaluation, transcatheter PVL closure was planned. Under general anesthesia and TEE/angio-fluoroscopic guidance, the PVL was successfully crossed via the right femoral artery, and a 10 mm × 4 mm Occlutech paravalvular leak device was deployed. Post-procedural imaging confirmed effective PVL closure with a trace-mild residual leak.

**Discussion:**

This case highlights the feasibility of transcatheter PVL closure as a less invasive alternative for patients with multiple prior SAVRs and high surgical risk. Advanced imaging techniques were crucial in procedural success, ensuring precise device placement. A multidisciplinary heart team approach is essential for optimizing outcomes in complex valve pathology. Long-term follow-up is necessary to monitor the durability of the closure and potential complications.

## Introduction

Paravalvular leaks (PVLs) are a known complication following surgical aortic (SAVR) and mitral (SMVR) valve replacements, as well as transcatheter aortic valve implantation (TAVI). They result from an incomplete seal between the prosthetic valve and native annulus due to tissue friability from infection (1) or calcification. PVLs are more common after SMVR (17%) than SAVR (10%) (2–5) and may appear intraoperatively or during follow-up. Late PVLs often stem from suture dehiscence due to prosthetic valve endocarditis (PVE) or residual annular calcifications (6). While 1%–5% of cases lead to heart failure (HF), hemolytic anemia (HA), or left ventricular enlargement (7), transcatheter PVL closure has become a less invasive alternative for high-risk surgical patients (8).

A double-chambered right ventricle (DCRV) is a rare congenital abnormality where hypertrophied muscle bundles divide the right ventricle into high- and low-pressure chambers. Typically diagnosed in childhood, a DCRV may persist into adulthood, often asymptomatically or mimicking other cardiovascular conditions, leading to misdiagnosis (9, 10).

## Case summary

A 46-year-old woman with hypertension and dyslipidemia was admitted with congestive heart failure [New York Heart Association (NYHA) class III] in late March 2005. Her history included a silent membranous ventricular septal defect (pmVSD) and severe symptomatic aortic regurgitation. In April 2005, she underwent bioprosthetic aortic valve replacement (Perimount #23, Edwards Lifesciences, USA), with concurrent pmVSD closure using 2-0 Ti-Cron™ polyester sutures. She showed significant clinical improvement with optimal medical therapy and regular follow-up.

Ten years later, the aortic valve prosthesis was replaced with a second bioprosthetic valve (Magna Ease #25, Edwards Lifesciences, USA) due to degeneration. In December 2018, she was hospitalized for sepsis caused by *Bordetella hinzii* and *Propionibacterium granulosum* infective endocarditis. A 45-day antibiotic regimen led to the normalization of inflammatory markers. Persistent infection suggested an antibiotic-resistant biofilm on the prosthetic valve (11).

In January 2019, she underwent a third SAVR with a mechanical aortic valve (On-X® #23, Life Technologies, Inc.). Surgical inspection revealed significant paravalvular leakage at the right coronary cusp and fibrous tissue (pannus) extending into the prosthesis orifice. Cultures confirmed *Acremonium* species infection.

**Figure 1:**
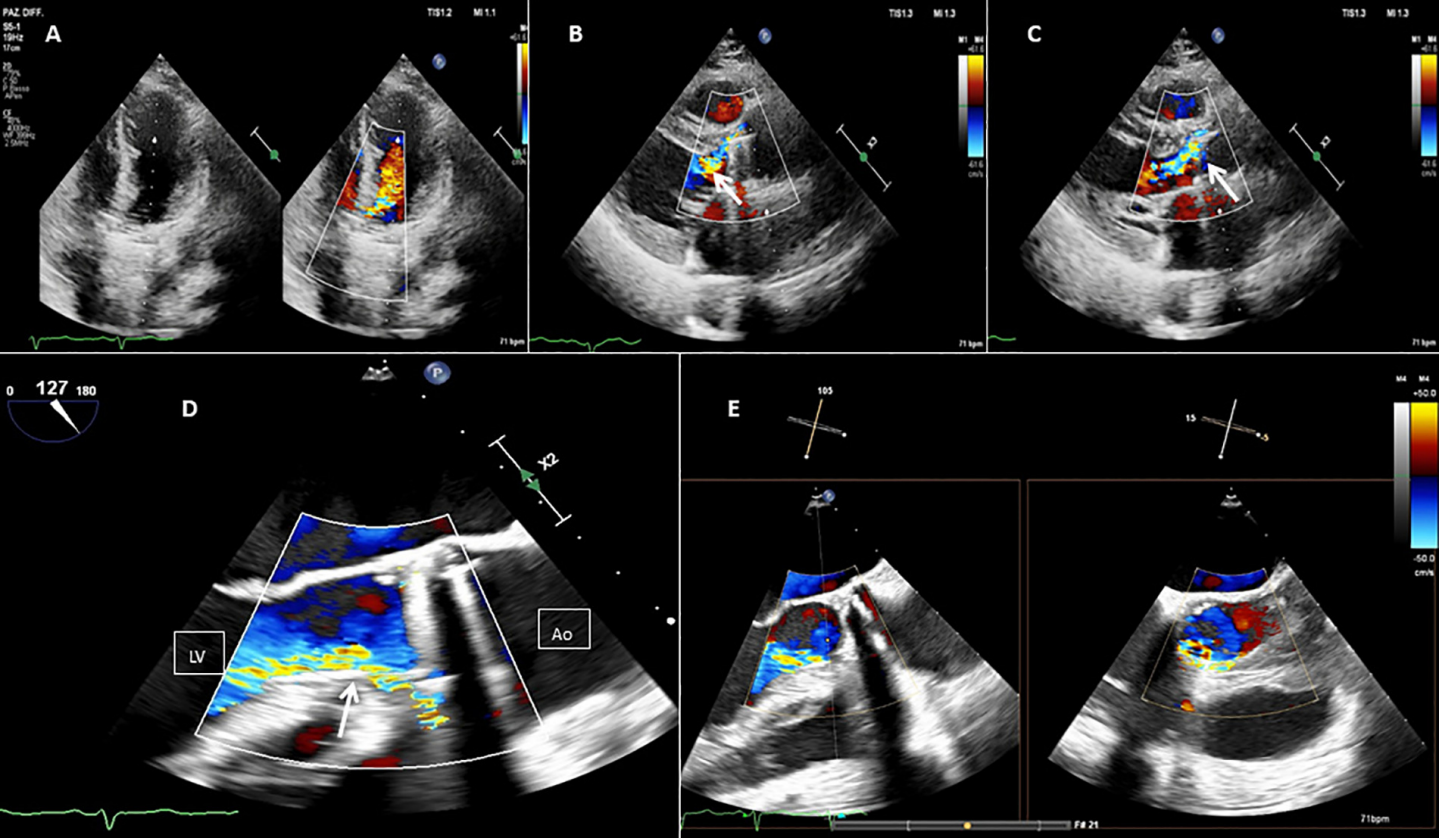
Baseline 2D TEE color Doppler at the apical five chamber view **(A)**, at the parasternal long axis view **(B,C)**, at the mid-esophageal (ME) long axis view **(D)**, and at the ME long axis view with Xplane **(E)**, showing an On-X® prosthetic aortic valve with moderate-to-severe regurgitation (orange arrow) through a long paravalvular leakage. Red line, PVL; LV, left ventricle; Ao, aortic valve.

**Figure 2:**
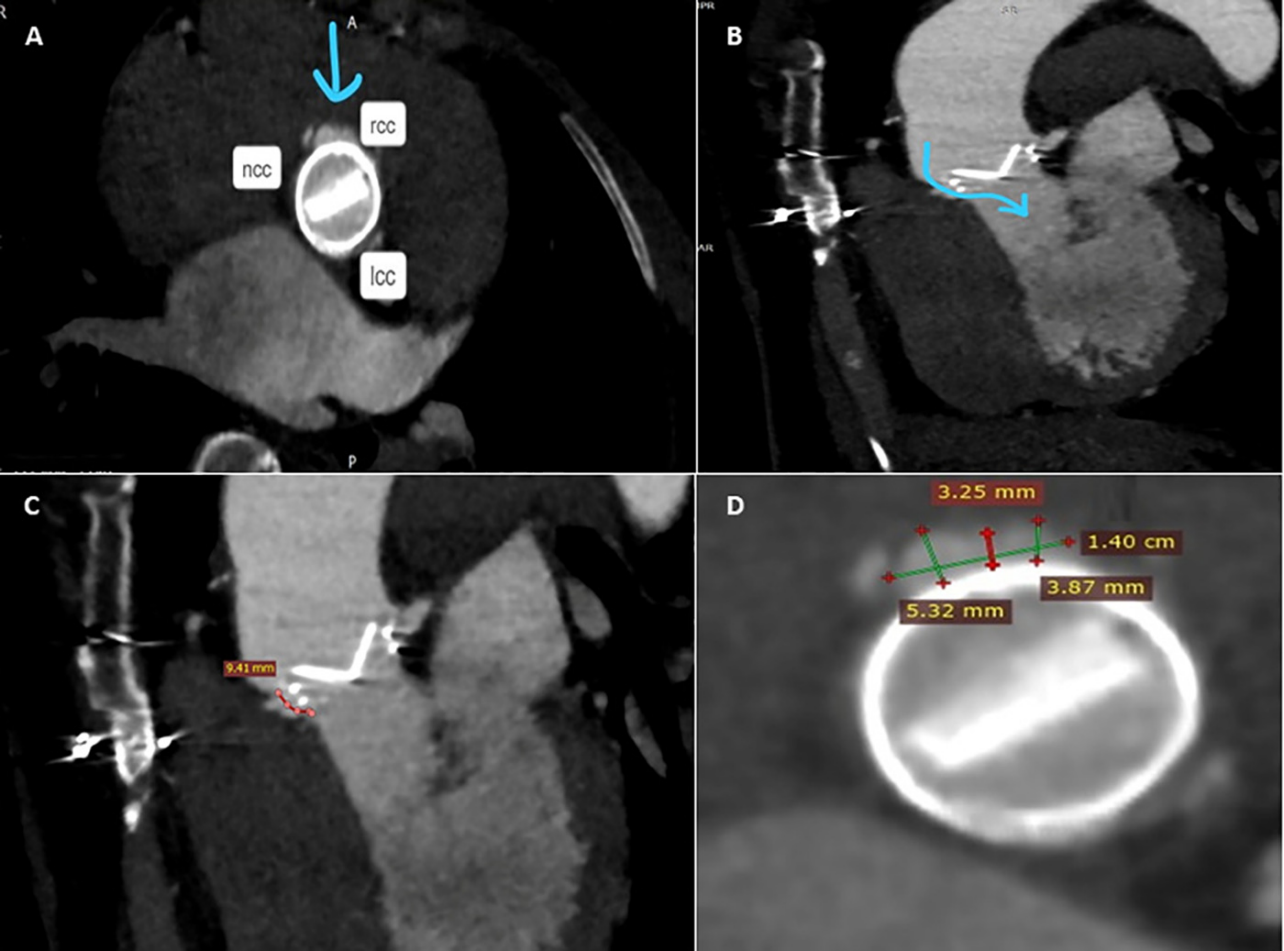
Pre-procedure MDCTA scans with acquired frames confirming the presence of the paravalvular leak, located in close proximity to the right coronary cusp **(A)**, and its channel configuration and length **(B,C)**. Short axis view of the aortic mechanical prosthetic valve showing multiple measurements of the long crescent-shaped PVL **(D)**. rcc, right coronary cusp; lcc, left coronary cusp; ncc, non-coronary cusp.

**Figure 3:**
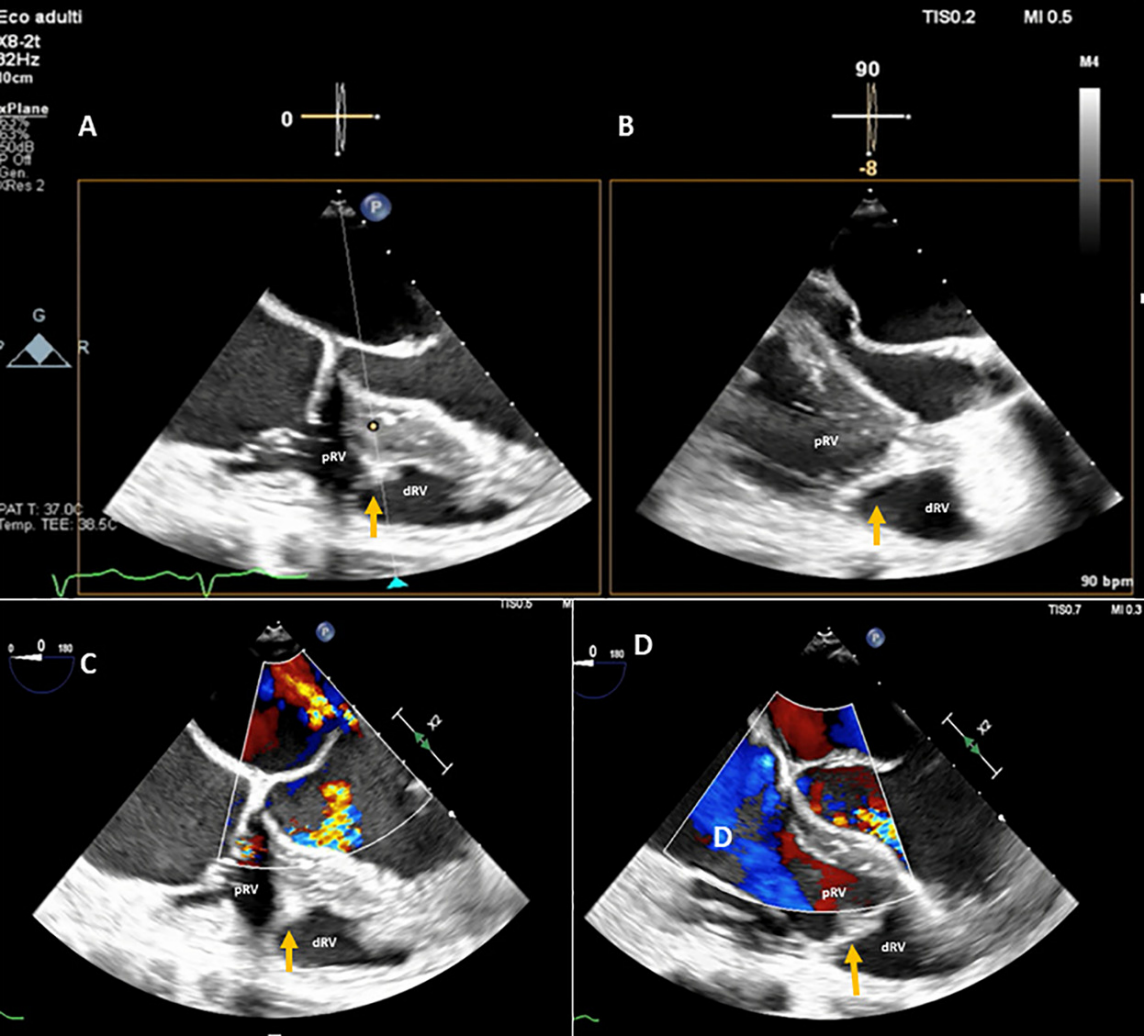
A DCRV created by an anomalous muscle band (orange arrow) diagnosed with TEE by the presence of a proximal high-pressure region under the tricuspid valve and a low-pressure distal region near the pulmonary valve outflow tract. **(A,B)** TEE transgastric RV inflow-outflow view (double orifice). **(C,D)** TEE color Doppler mid-esophageal four-chamber view focused on the RV. pRV, proximal right ventricle; dRV, distal right ventricle.

Subsequent imaging (2019–2021) showed mild residual PVL regurgitation with normal valve function. However, by 2024, two-dimensional transesophageal echocardiography (2D TEE) and multidetector computed tomography angiography (MDCTA) confirmed moderate-severe PVL regurgitation (vena contracta width 0.6 cm) near the right coronary cusp (Figures 1, 2). A coincidental DCRV was also identified (Figure 3, Supplementary Video 1). Given the prohibitive surgical risk, transcatheter PVL closure was planned.

**Figure 4:**
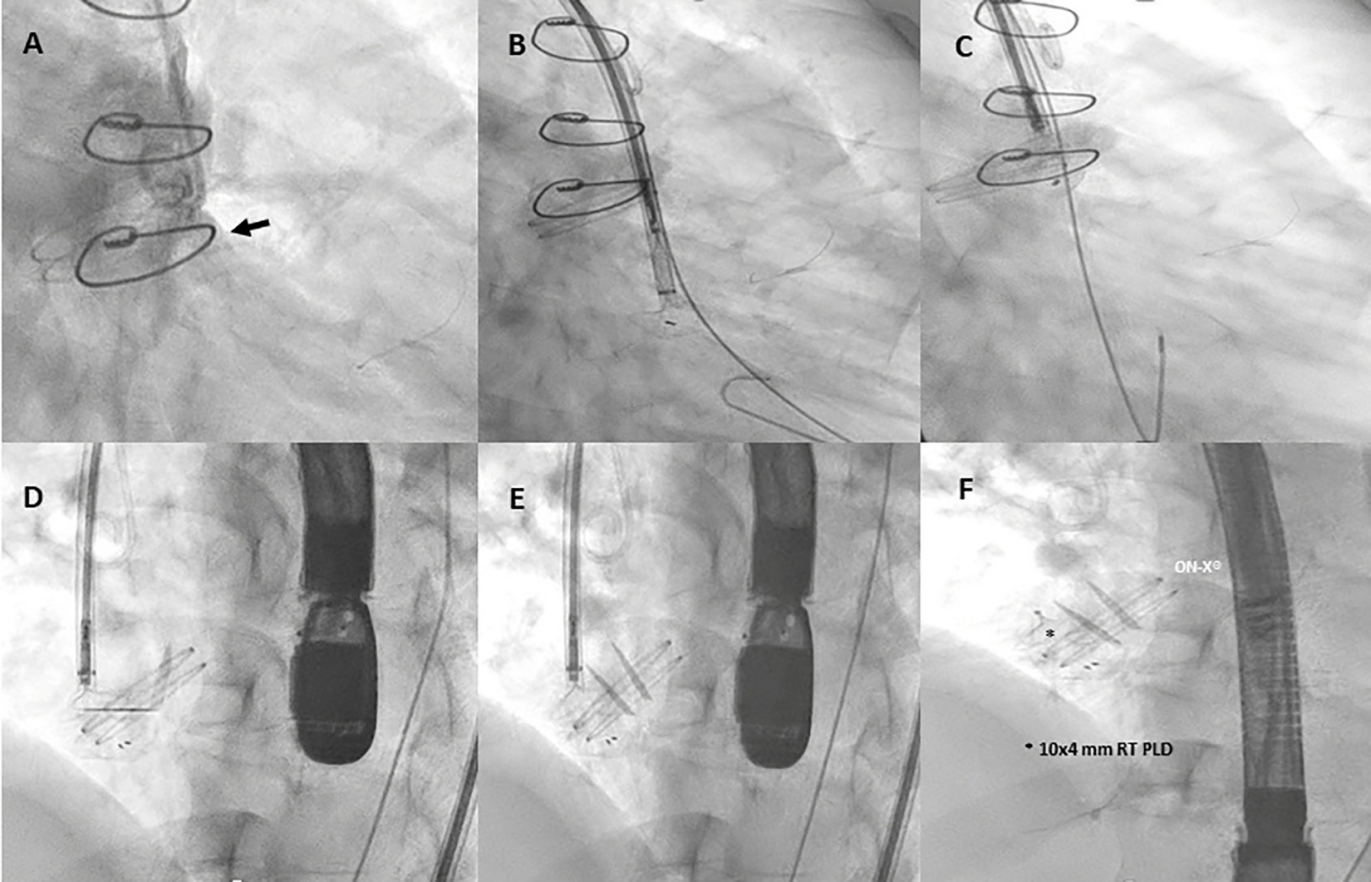
Fluoro-angiography procedural steps. **(A)** Right anterior oblique aortography by pigtail 6-Fr showing PVL regurgitation (black arrow). **(B,C)** The left disc of a 10 mm × 4 mm rectangular twist (RT) PLD (Occlutech, Helsingborg, Sweden) is opened into the left ventricle through an 8-Fr dedicated delivery sheath (Occlutech ODS III) across the leakage, with a buddy wire (black arrowhead) at its site; **(D–F)** 10 mm × 4 mm RT PLD successfully deployed and released within the leak without interfering with normal leaflet movement of the On-X® prosthetic aortic valve.

The procedure was performed in March 2024 under general anesthesia with TEE and angio-fluoroscopic guidance. A hydrophilic guidewire crossed the PVL and was replaced with an Amplatz Super Stiff™ wire. An 8-Fr delivery sheath was advanced, and a 10 mm × 4 mm Occlutech paravalvular leak device (PLD) was successfully deployed, achieving significant leak reduction without prosthetic interference (Figure 4, Supplementary Figure 1). Post-procedure imaging confirmed device stability with a trace-mild residual leak (Supplementary Figure 2). The patient was discharged on postoperative day 3 in better clinical conditions.

Finally, 3- and 12-month follow-ups confirmed clinical improvement, enhanced quality of life, and device stability with a persistent trace-mild residual leak.

## Discussion

PVE accounts for 20% of infective endocarditis cases, leading to valve dysfunction, heart failure, and increased mortality (12, 13). PVE is more frequent in bioprosthetic valves, particularly porcine, due to susceptibility to bacterial colonization (14–16). Our case of *B. hinzii* and *P. granulosum* endocarditis is rare. *B. hinzii*, an emerging Gram-negative pathogen, is often resistant to antibiotics, while *P. granulosum* requires prolonged culture incubation for detection (17, 18).

These device-related microorganisms can be difficult to eradicate by antibiotic therapy alone. Bacterial biofilms are communities of various microorganisms that adhere to any surface and are enveloped within extracellular polymeric substances (19). Disruption of the bacterial biofilm plays an essential role in recovering the causative agent in the culture. Certain culture and nucleic acid amplification techniques are more accurate in guiding directed treatment regimens. It is clear that diagnostic testing will need to evolve beyond the standard current microbiological procedures to include nucleic acid detection, enhanced culture techniques, novel microbe imaging, and local immune response (20).

Transcatheter PVL closure is a minimally invasive alternative for high-risk patients who are often ineligible for redo surgery (21). PVLs are very heterogeneous in their location, size, and shape, and their track can be tortuous, serpiginous, or heavily calcified.

Undoubtedly, aortic PVL closure is a complex and challenging procedure with several periprocedural complications, such as device malposition or embolization and residual regurgitation, requiring optimal patient selection and comprehensive intraprocedural imaging guidance. Furthermore, a successful PVL reduction results in acute and long-lasting symptomatic improvements in clinical parameters and NYHA class, reducing the need for repeat cardiac surgeries and blood transfusions (22).

PVL closure has been performed in the past with multiple off-label devices that were not specifically designed for the purpose, which had inappropriate designs, shapes, and sizes. In previous studies with off-label devices, technical success rates varied between 62% (23) and 87% (5).

Notably, the Occlutech PLD, designed specifically for PVL closure, offers improved stability and sealing, achieving procedural success in over 90% of aortic PVL cases (24).

DCRV, a rare congenital defect (0.5%–2% of congenital heart disease), typically presents in childhood but can persist asymptomatically in adults (25–29). It results from hypertrophic muscle bands dividing the right ventricle into high- and low-pressure chambers. Though often misdiagnosed, our patient remained asymptomatic with a low-pressure gradient, requiring only medical follow-up.

## Conclusion

This case highlights the effectiveness of transcatheter aortic PVL closure as a safe and less invasive alternative for high-risk patients with recurrent infections. The concomitant asymptomatic DCRV required no intervention.

## Data Availability

The raw data supporting the conclusions of this article will be made available by the authors, without undue reservation.

## Supplementary material

Supplementary Figure 1 Real-time three-dimensional transoesophageal echocardiography at aortic view showing the 10x4 mm rectangular twist (RT) PLD (Occlutech, Helsingborg, Sweden) in situ.

Supplementary Figure 2 Post-procedure TTE color Doppler parasternal long axis view showing trace-mild residual leak. Ao, aorta; LV, left ventricle; LA, left atrium.

Supplementary Video 1 Two-dimensional (2D) transesophageal echocardiography (TEE) color Doppler at transgastric RV inflow-outflow view (double orifice) **(A)** and TEE mid-esophageal (ME) short axis view **(B)** focused on right ventricle illustrating double-chambered right ventricle (DCRV) created by anomalous muscle band (orange arrow) with the presence of a proximal high-pressure region under the tricuspid valve and a low-pressure distal region near the pulmonary valve outflow tract. pRV, proximal right ventricle; dRV, distal right ventricle.
