# Supplementary material for: Case Report: Moderate-to-severe paravalvular leak regurgitation after recurrent prosthetic valve endocarditis in a patient with a double-chambered right ventricle associated with a restricted membranous ventricular septal defect
Source: Front Cardiovasc Med. 2025 May 14;12:1558686. doi: 10.3389/fcvm.2025.1558686 (PMC12116576; doi:10.3389/fcvm.2025.1558686)
Supplement: Supplementary Figure 1 — Real-time three-dimensional transoesophageal echocardiography at aortic view showing the 10x4 mm rectangular twist (RT) PLD (Occlutech, Helsingborg, Sweden) in situ. [file Datasheet1.pdf]

TIMELINE

April 2005

- Congestive heart failure (NYHA III) due to severe aortic regurgitation
- SAVR with a bioprosthetic aortic valve (**Perimount # 23 mm**) with concurrent pmVSD closure using 2-0 Ti-Cron™ polyester sutures

May 2015

- Aortic valve prosthesis was replaced with a second bioprosthetic valve (**Magna Ease #25**) due to degeneration

December 2018

- Hospitalization for sepsis caused by Bordetella Hinzii and Propionibacterium granulosum infective endocarditis.
- A 45-day antibiotic regimen led to the normalization of inflammatory markers. Persistent infection suggested an antibiotic-resistant biofilm on the prosthetic valve.

January 2019

- Third SAVR, this time with a mechanical aortic valve (**On-X® #23**).
- Surgical inspection revealed significant paravalvular leakage at the right coronary cusp and fibrous tissue (pannus) extending into the prosthesis orifice.

2019-2021

- Subsequent imaging showed mild residual PVL regurgitation with normal valve function. A double-chambered right ventricle (DCRV) was also identified. coincidental, asymptomatic

January 2024

- 2D TEE and MDCTA confirmed moderate-severe PVL regurgitation (vena contracta width 0.6 cm) near the right coronary cusp.
- Given the prohibitive surgical risk, transcatheter PVL closure was planned.

March 2024

- A 10x4 mm Occlutech Paravalvular Leak Device (PLD) was successfully deployed, achieving significant leak reduction without prosthetic interference.
- Post-procedure imaging confirmed device stability with a trace to mild residual leak.

April 2024

- 12-month follow-up confirmed clinical improvement and enhanced quality of life, device stability with a persisting trace-mild residual leak.
