# Supplementary figures and images for: Case Report: Moderate-to-severe paravalvular leak regurgitation after recurrent prosthetic valve endocarditis in a patient with a double-chambered right ventricle associated with a restricted membranous ventricular septal defect
Source: Front Cardiovasc Med. 2025 May 14;12:1558686. doi: 10.3389/fcvm.2025.1558686 (PMC12116576; doi:10.3389/fcvm.2025.1558686)

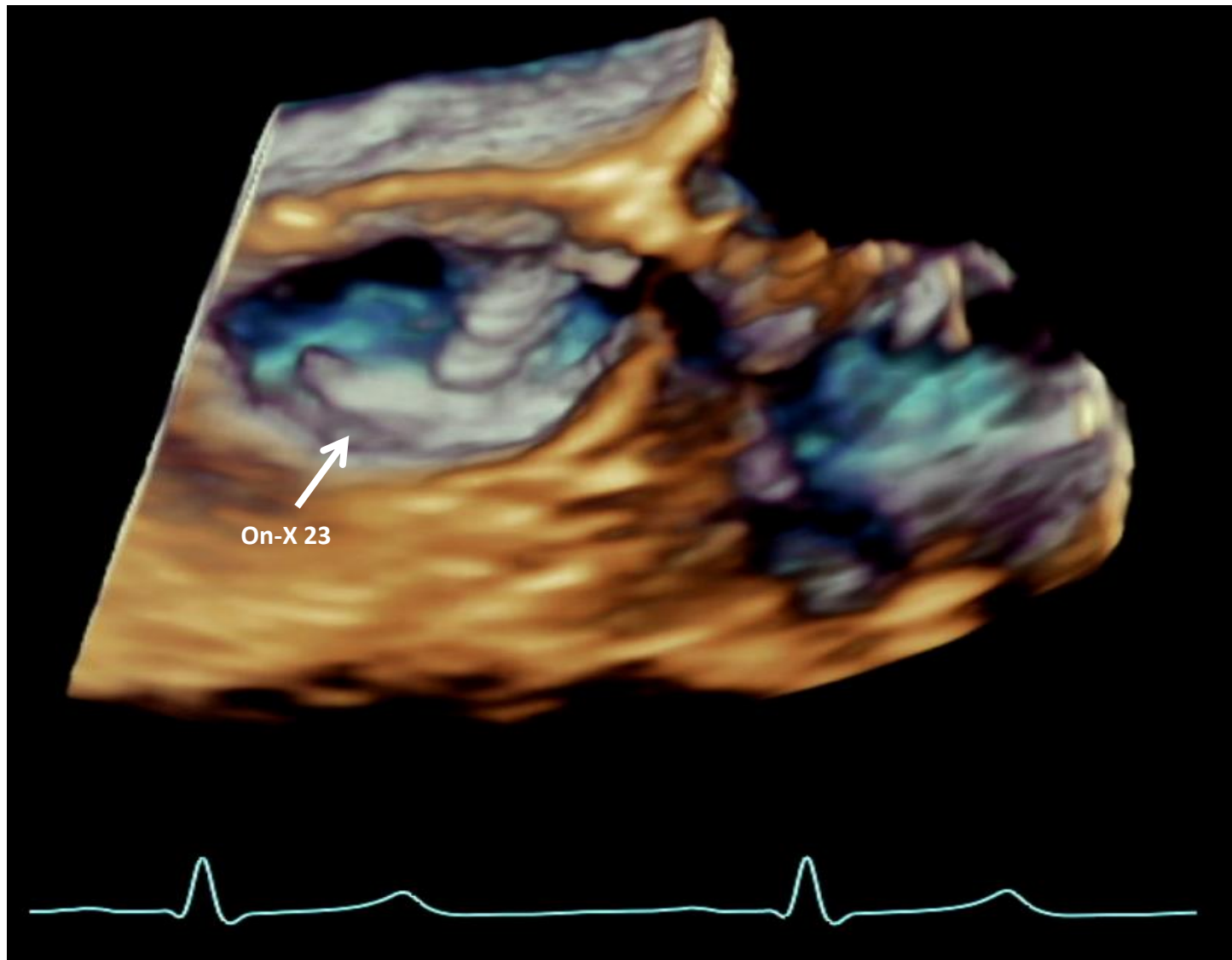

Supplementary Figure 1

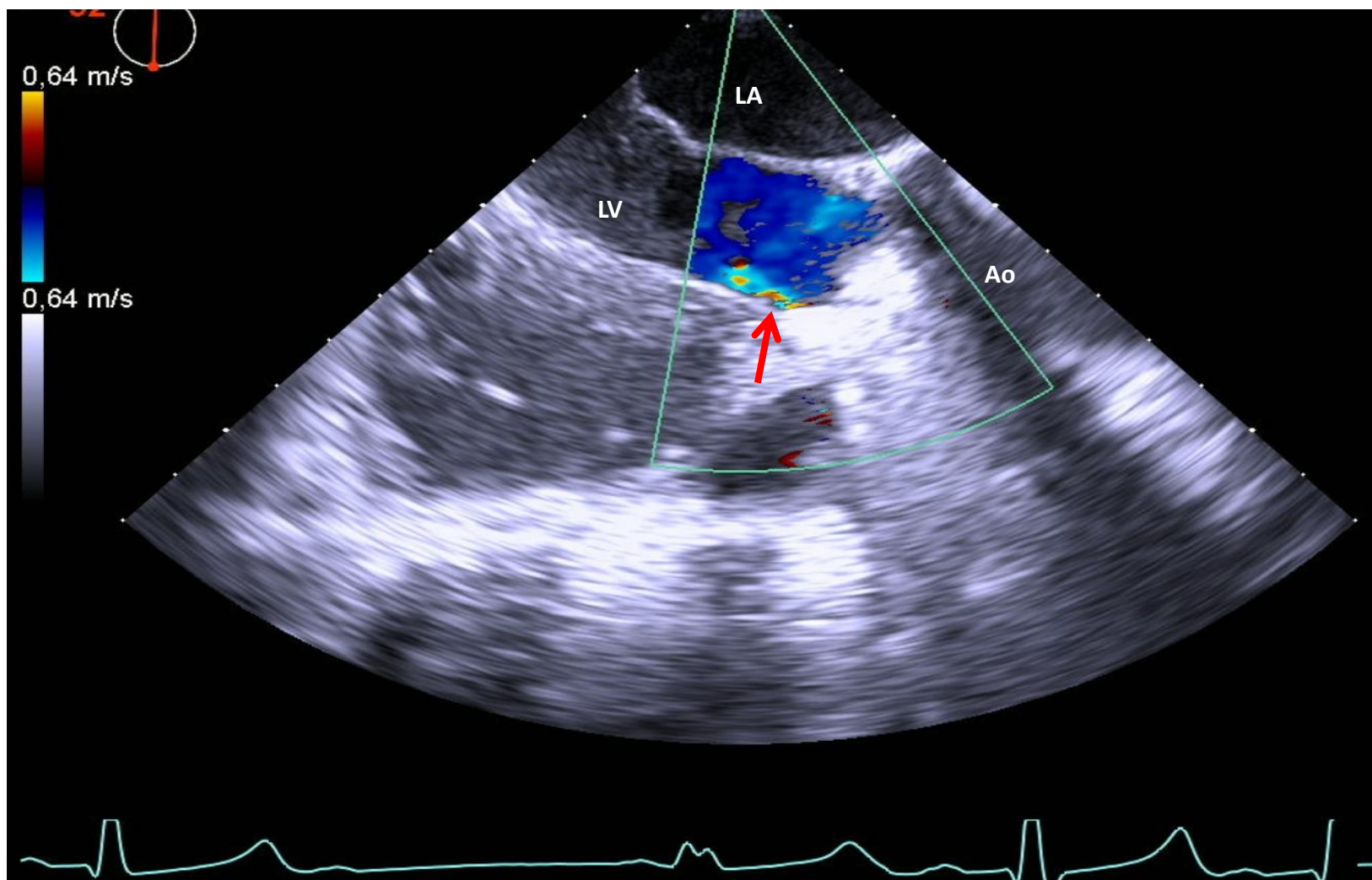

Supplementary Figure 2

Supplement: Supplementary Figure 2 — Post-procedure TTE color Doppler parasternal long axis view showing trace-mild residual leak. Ao, aorta; LV, left ventricle; LA, left atrium. [file Datasheet2.pdf]

## Slide 1
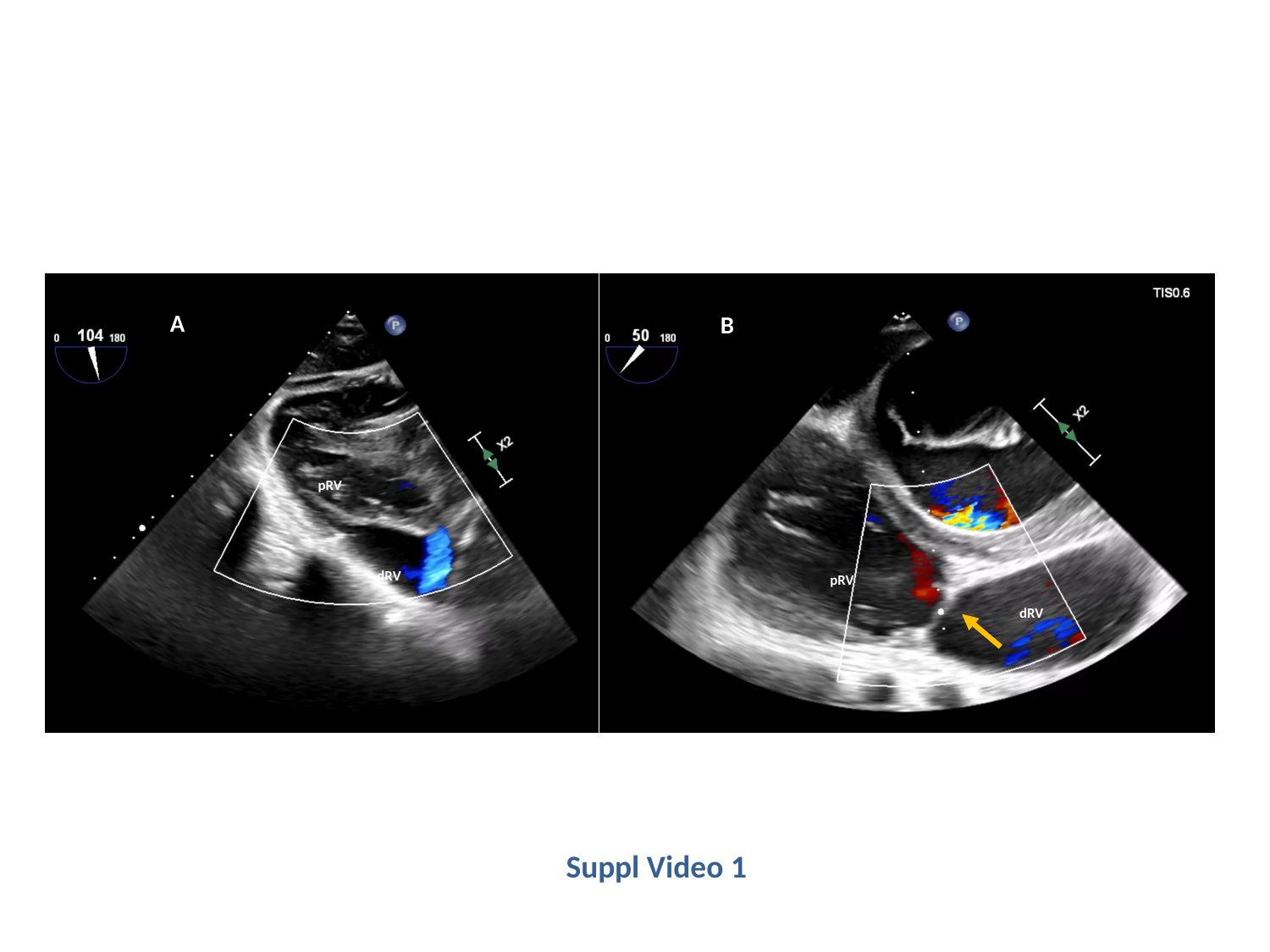

A
B
pRV
dRV
pRV
dRV
Suppl Video 1
LA

Supplement: Supplementary Video 1 — Two-dimensional (2D) transesophageal echocardiography (TEE) color Doppler at transgastric RV inflow-outflow view (double orifice) (A) and TEE mid-esophageal (ME) short axis view (B) focused on right ventricle illustrating double-chambered right ventricle (DCRV) created by anomalous muscle band (orange arrow) with the presence of a proximal high-pressure region under the tricuspid valve and a low-pressure distal region near the pulmonary valve outflow tract. pRV, proximal right ventricle; dRV, distal right ventricle. [file Presentation1.pptx]
